# A general toxicity and biodistribution study of human natural killer cells by single or repeated intravenous dose in severe combined immune deficient mice

**DOI:** 10.1007/s43188-022-00138-0

**Published:** 2022-06-13

**Authors:** Sang-Jin Park, Hae-Jin Yoon, Eun-Young Gu, Byoung-Seok Lee, Yongman Kim, Jaeseob Jung, Jinmoon Kim, Kyoung-Sik Moon

**Affiliations:** 1 Department of Toxicological Evaluation and Research, Korea Institute of Toxicology, 141 Gaejeongro, Yuseong gu, Daejeon, Republic of Korea; 2 NKMAX Co., Ltd, SNUH Healthcare Innovation Park, Seongnam, Gyeonggi-do Republic of Korea

**Keywords:** Natural killer cells, Toxicity, Biodistribution, Cell therapy

## Abstract

Natural killer (NK) cells are a part of the innate immune system and represent the first line of defense against infections and tumors. NK cells can eliminate tumor cells without major histocompatibility restriction and are independent of the expression of tumor-associated antigens. Therefore, they are considered an emerging tool for cancer immunotherapy. However, the general toxicity and biodistribution of NK cells after transplantation remain to be understood. This study was conducted to evaluate the general toxicity and biodistribution of human NK cells after single or repeated intravenous dosing in severely combined immunodeficient (SCID) mice. There were no test item-related toxicological changes in single and repeated administration groups. The no observed adverse effect level of human NK cells was 2 × 10^7^ cells/head for both male and female SCID mice. Results from the biodistribution study showed that human NK cells were mainly distributed in the lungs, and a small number of the cells were detected in the liver, heart, spleen, and kidney of SCID mice, in both the single and repeated dose groups. Additionally, human NK cells were completely eliminated from all organs of the mice in the single dose group on day 7, while the cells persisted in mice in the repeated dose group until day 64. In conclusion, transplantation of human NK cells in SCID mice had no toxic effects. The cells were mainly distributed in the lungs and completely disappeared from the body over time after single or repeated intravenous administration.

## Introduction

Natural killer (NK) cells are part of the innate immune system, as a subset of peripheral blood lymphocytes that represents the first line of defense against infections and tumors [1]. NK cells lyse tumor cells without prior sensitization and are independent of major histocompatibility (MHC) restriction, and they secrete several cytokines to initiate adaptive immune responses against tumors [2]. NK cells are the most potent cytotoxic lymphocytes and play an important role in suppressing tumor metastasis and outgrowth using a variety of effector mechanisms, including the perforin/granzyme-containing granule-mediated pathway, death-receptor pathway, and interferon-γ-mediated pathway [3, 4]. NK cell-based immunotherapies are an attractive approach because NK cells can eliminate tumor cells in a non-MHC-restricted manner and are independent of the expression of tumor-associated antigens [5, 6].

Expansion of NK cells ex vivo is necessary to obtain sufficient cell numbers for adoptive transfer, as these cells represent low frequency in peripheral blood and cord blood cells and have limited lifespans [7–9]. However, NK cells therapies processed ex-vivo may result in unexpected risks because the expansion and preservation process could be affected by cytokines and interleukins. In addition, several recent studies have reported that NK cells boost anti-tumor function, support *in vivo* persistence and homeostatic proliferation, and promote homing to the tumor microenvironment [10–12].

Given the increasing challenges related to NK cell immunotherapy against tumors, information on the safety assessment of NK cells after transplantation needs to be understood for subsequent studies in humans. To date, there are few literatures documenting the toxicity and biodistribution profile of human NK cells in preclinical studies. This study was conducted to evaluate the general toxicity and biodistribution of human NK cells, which were produced under good manufacturing practice (GMP) conditions, after single or repeated intravenous dosing in severely combined immunodeficient (SCID) mice.

## Materials and methods

### Isolation and characterization of human NK cells

#### NK cell enrichment and expansion

All manufacturing and product testing procedures for the generation of human NK cells were performed under GMP conditions (NKMAX Co., Ltd, Seongnam, Korea). Peripheral blood mononuclear cells (PBMCs) were isolated from healthy donors by leukapheresis, and NK cells were expanded as described previously [13], with some modifications. Briefly, CD56^+^ cells were isolated from PBMCs using CliniMACS CD56 microbeads (Miltenyi Biotech GmbH, Galdbach, Germany) according to the manufacturer’s instructions. Isolated CD56^+^ cells were then cultured in RPMI-1640 medium (WELGENE Inc., Gyeongsan, Korea) supplemented with 10% fetal bovine serum (FBS; Hyclone, Tauranga, New Zealand), 20 µg/mL gentamicin (GIBCO, Grand Island, NY), g-irradiated (100 Gy) KL-1 and LCL feeders, 500 IU/mL IL-2 (PROLEUKIN^®^; Norvatis, Basel, Switzerland), and 50 ng/mL IL-21 (NKMAX Co., Ltd., Seongnam, Korea). Growing NK cells were sub-cultured every 3–4 days with fresh RPMI-1640 medium containing IL-2. After 17–18 days of culture, cells were harvested and washed.

#### Immunostaining and flow cytometric analysis

The following monoclonal antibodies were used to stain NK cells: anti-CD56-FITC, anti-CD3-PE, anti-CD20-PerCP-Cy5.5, and anti-CD14-APC (BD Biosciences). NK cells were stained with the antibodies for 30 min at 4 °C. Sample data were acquired on a FACS flow cytometer (BD FACSDiva™) and analyzed using BD FACSuite v1.2 software.

#### Cytotoxicity assay

The cytotoxicity of NK cells against K562 cells was assessed using a fluorometric cytotoxicity assay. K562 cells were stained with 4 mM calcein-AM solution (Sigma) for 30 min at 37 °C. NK cells and target cells were mixed at an E:T ratio of 10:1 and cocultured in 96-well U-bottom plates. After 4 h of incubation, 80 µL of the supernatant was transferred to a new 96-well flat-bottom plate. Fluorescence signals were determined using a SpectraMax M2 microplate reader (Molecular Devices, San Jose, CA, USA) and excited at 485 nm, and emission was detected at 525 nm. The percent specific lysis was calculated using the following formula: [(test release − spontaneous release)/(maximum release − spontaneous release)] × 100.

### Experimental designs

#### Experimental animals and husbandry

Male and female SCID mice (5–6 weeks old) were purchased from Charles River Laboratories Japan (Kanagawa, Japan). Animals were selected for use in the study on the basis of adequate body weight and freedom from clinical signs of disease or injuries. The mice were inoculated with human NK cells at approximately 6–7 weeks after the acclimation period. The animals were assigned to treatment groups in a stratified manner, using the Pristima System (Version7.2, or 7.3 Xybion Medical System Co., USA), based on the most recent body weight. The animal room environment was automatically controlled (target range: temperature 23 ± 3 °C, relative humidity 30–70%, approximately 12 h light cycle with 150–300 lx, and ventilation 10–20 times/h). A standard mouse pellet diet was provided ad libitum. Microbial monitoring for diet was performed, and a certificate of analysis for the diet was provided by the source. The animals had ad libitum access to filtered, ultraviolet light-irradiated municipal tap water at all times. All animal experiments were conducted under good laboratory practice conditions and reviewed and assessed by the Institutional Animal Care and Use Committee of the Korea Institute of Toxicology (Table 1).

**Table 1:** Hematology of male and female mice after administration of NKMAX Cell Therapy Product

Parameter	Male SCID mice					Female SCID mice				
	VC	5 × 10^6^	2 × 10^7^	VC (R)	2 × 10^7^ (R)	VC	5 × 10^6^	2 × 10^7^	VC (R)	2 × 10^7^ (R)
MONA (%)	0.12 ± 0.036	0.11 ± 0.058	0.12 ± 0.044	0.08 ± 0.012	0.08 ± 0.032	0.11 ± 0.025	0.09 ± 0.014	0.06 ± 0.019^*D^	0.19 ± 0.007	0.18 ± 0.055
RET (%)	2.5 ± 0.216	2.55 ± 0.341	2.53 ± 0.325	2.99 ± 0.249	2.84 ± 0.159	2.51 ± 0.44	2.79 ± 0.249	2.67 ± 0.757	2.19 ± 0.113	2.99 ± 0.303^*D^
RETA (× 10^9^/L)	264 ± 25.15	268 ± 41.21	264.4 ± 30.87	319.4 ± 30.75	299 ± 14.54	261.9 ± 45.62	283.7 ± 23.41	279.8 ± 82.18	239.2 ± 17.82	323.8 + 28.47^*D^

Only parameters with statistical significance were described

*R* recovery period, *RET* relative reticulocytes counts, *RETA* Absolute reticulocytes counts, *SCID* severely combined immunodeficient, *VC* vehicle control

^*R^Dunn Rank Sum test significant at the 0.05 level

^*D^Dunnet LSD test significant at the 0.05 level

#### Single dose toxicity study

Both male and female mice were randomly assigned to the following four groups, consisting of five animals/group: vehicle control (VC), low-dose (5 × 10^6^ cells/head), medium dose (1 × 10^7^ cells/head), and high-dose (2 × 10^7^ cells/head) groups. Mice in the VC group were inoculated with Hartmann solution, containing IL-2. Mice were inoculated intravenously once via the tail vein, and mortality, clinical signs, body weight, and macroscopic observations were evaluated for 2 weeks following a single intravenous injection.

#### Eight weeks repeated dose toxicity study

A preliminary study (2 weeks repeated dose toxicity study) was conducted to investigate the approximate toxicity of human NK cells after 10 repeated intravenous administrations, five times a week for 2 weeks, and to evaluate the reversibility for 4 weeks. Considering cell viability and potency, human NK cells can be formulated to contain up to 1 × 10^8^ cells/mL. Therefore, 2 × 10^7^ cells/0.2 mL/head was selected because it is the maximum dose that can be intravenously injected into mice. For 8 weeks repeated dose toxicity study, ten male and ten female animals were assigned to the main group, and six male and six female animals were assigned to the recovery group. At a dose levels of 0 (VC group), 5 × 10^6^ and 2 × 10^7^ cells/head were administered three times a week for 8 weeks. The animals were observed for mortality, clinical signs, body weight, food consumption, ophthalmology, hematology, clinical chemistry, urinalysis, macroscopic findings, organ weights, and microscopic findings.

#### Real-time polymerase chain reaction (qPCR) method of validation for biodistribution

A qPCR method designed to detect human-specific *Alu* gene for biodistribution study was validated. The validation parameters studied according to the test guidelines of the Ministry of Korean Food and Drug Safety were linearity, specificity, sensitivity (limit of detection), accuracy, and precision.

#### Biodistribution analysis

A biodistribution study was conducted to measure the human-specific *Alu* gene of human NK cells using qPCR analysis and was performed according to the validated method. Five male and five female animals were intravenously administered single or multiple doses (once a week for 6 weeks) of human NK cells (2 × 10^7^ cells/head) and were maintained until the scheduled sacrifice day [day 0 (1 h after administration), day 1, day 7, day 14, day 28, day 35, day 42, day 49, and day 63]. After all animals were necropsied, the major organs (heart, lung, liver, spleen, kidney, testis/ovary, epididymis, uterus with cervix, brain, mesenteric lymph node, skeletal muscle, and adrenal gland) were harvested, frozen in liquid nitrogen, and ground in an automatic homogenizer Precellys 24 (Bertin Technologies, Aix en Provence, France Chiba, Japan) for DNA extraction, using a Maxwell 16 instrument (Promega, WI, USA). DNA was extracted on the day of euthanasia. The concentration of DNA was measured using a SPECTROstar Nano spectrophotometer (BMG Labtech, Offenburg, Germany). The *Alu* gene was amplified from the extracted DNA using qPCR, with the following primers: sense-GTCAGGAGATCGAGACCATCCC, and anti-sense-TCCTGCCTCAGCCTCCCAAG [14]. qPCR was performed using the QuantStudio 5 Real-Time PCR System and ViiA7 Real-Time PCR System (Thermo Fisher Scientific, MA, USA), using the following conditions: 95 °C for 2 min followed by 35 cycles of 95 °C for 15 s, 68 °C for 30 s, and 72 °C for 30 s. A melt curve was obtained after each qPCR to ensure the precise amplification of the amplicons. This consisted of 20 s at 72 °C, followed by a stepwise increase of 1 °C with a 5-s hold at each step.

### Animal observation

#### Mortality and clinical signs

Mortality and moribundity were observed twice a day, and clinical signs, including general appearance and behavioral changes, were recorded.

#### Body weight and food consumption

Body weight of the mice was measured at receipt, grouping, and approximately once a week during the treatment period. The amount of food in each cage was measured. The quantity of food consumed by every animal in each cage, 2 days during the whole week (5–7 days), was measured and presented as g/animal/day.

#### Ophthalmological examinations

Ophthalmological examinations were performed on all animals in the repeated toxicity studies, using binocular indirect ophthalmoscopy (Vantage Plus Digital, Keeler Ltd., England), after the animals were treated with mydriatics (Mydrin-P, Santen Pharmaceutical Co., Japan).

### Laboratory investigations

#### Hematology

Animals were fasted overnight prior to necropsy and sacrificed after blood collection, under isoflurane anesthesia, and EDTA-2 K was used as an anticoagulant. The following parameters were analyzed: white blood cell count, red blood cell count, hemoglobin concentration, hematocrit, mean corpuscular volume, mean corpuscular hemoglobin, mean corpuscular hemoglobin concentration, platelets, neutrophils, lymphocytes, monocytes, eosinophils, basophils, large unstained cells, and reticulocytes. This was done using a hematological autoanalyzer (ADVIA2120i Hematology analyzer, Bayer, USA).

#### Serum biochemistry

Serum was isolated and used to determine the levels of glucose, alanine aminotransferase, blood urea nitrogen, aspartate aminotransferase, creatinine, total bilirubin, total protein, alkaline phosphatase, albumin, gamma glutamyl transpeptidase, albumin/globulin ratio, creatine phosphokinase, total cholesterol, calcium, triglyceride, and inorganic phosphorus; this was done using a clinical chemistry auto-analyzer (200FR NEO, Toshiba Co., Japan).

#### Urinalysis

Urine samples were collected at approximately 3–4 h within 1 week prior to necropsy. The following parameters were measured during urinalysis: color, clarity, glucose, pH, erythrocyte, specific gravity, ketone, bilirubin, leukocytes, protein, nitrite, and urobilinogen. All parameters except for urine color were analyzed using Combur 10 TM urine sticks (Roche, Germany) and a Cobas U411 urine analyzer (Roche, Germany).

### Necropsy

Animals were sacrificed via exsanguination of the aorta and posterior vena cava under isoflurane anesthesia. Complete necropsy examinations were performed on all animals that were found dead or were terminally sacrificed under the direct supervision of a veterinary pathologist. After blood sampling, the animals were subjected to necropsy and carefully examined for external abnormalities (Table 2).

**Table 2:** Biochemistry of male and female mice after administration of NKMAX Cell Therapy Product

Parameter	Male SCID mice					Female SCID mice				
	VC	5 × 10^6^	2 × 10^7^	VC (R)	2 × 10^7^ (R)	VC	5 × 10^6^	2 × 10^7^	VC (R)	2 × 10^7^ (R)
ALB (g/dL)	3.53 ± 0.097	3.37 ± 0.182	3.28 ± 0.052^*D^	3.41 ± 0.075	3.50.22	3.43 ± 0.117	3.55 ± 0.128	3.44 ± 0.183	3.66 ± 0.145	3.28 ± 0.283
AST (IU/L)	63.7 ± 10.41	57 ± 8.51	59.6 ± 6.14	46.6 ± 1.24	49.9 ± 1.01^*D^	70.3 ± 5.9	67.7 ± 7.61	86.6 ± 51.22	66.5 ± 6.7	57.9 ± 7.85
TCHO (mg/dL)	150.6 ± 11.84	134 ± 5.79^*D^	134.5 ± 9.15^*D^	130.7 ± 4.51	136.3 ± 8.5	93.8 ± 3.35	95.2 ± 7.79	104.4 ± 13.35	107.7 ± 9.29	115.5 ± 9.19
GGT (IU/L)	0.88 ± 0.281	0.63 ± 0.352	0.73 ± 0.219	1.44 ± 0.208	1.29 ± 0.151	0.45 ± 0.445	0.09 ± 0.108	1.22 ± 0.321^+D^	1.51 ± 0.095	1.26 ± 0.191

Only parameters with statistical significance were described

*ALB* albumin, *AST* aspartate transaminase, *GGT* gamma glutamyl transpeptidase, *R* recovery period, *SCID* severely combined immunodeficient, *TCHO* total cholesterol, *VC* vehicle control

^*R^Dunn Rank Sum test significant at the 0.05 level

^+R^Dunn Rank Sum test significant at the 0.01 level

^*D^Dunnet LSD test significant at the 0.05 level

^+D^Dunnet LSD test significant at the 0.01 level

### Organ weights

The weights of the brain, pituitary gland, liver (with gall bladder), spleen, heart, salivary glands (submandibular and sublingual), seminal vesicles (with coagulation glands), prostate, kidneys, adrenal glands, testes, epididymides, lungs, thyroids (with parathyroids), thymus, uterus (with cervix), and ovaries were measured for all animals at terminal and recovery sacrifice, and organ/brain and body weight ratios (using the terminal body weight obtained prior to necropsy) were calculated. Bilateral organs were measured together.

### Histopathology

Tissue samples from adrenal glands, aorta (thoracic), brain, cecum, colon, duodenum, esophagus, femur with marrow (F-T joint), heart, ileum, jejunum, kidneys, liver (with gallbladder), lung (with bronchi), mammary gland (female only), mandibular lymph node, mesenteric lymph node, ovaries, pancreas, pituitary gland, prostate, rectum, salivary glands (submandibular and sublingual), sciatic nerve, seminal vesicles (with coagulation gland), skeletal muscle, skin (inguinal), spinal cord (cervical, thoracic, lumbar), spleen, sternum (with marrow), stomach, thyroids (with parathyroids), thymus, tongue, trachea, urinary bladder, uterus with cervix, vagina, and injection site (tail) were preserved in 10% neutral buffered formalin, while the eyes (with optic nerve) were fixed in Davidson’s fixative and the testes and epididymides were fixed in Bouin’s fixative. The tissues were placed in the appropriate fixative for approximately 48 h and then transferred to 70% ethanol. Formalin was infused into the lungs via the trachea and into the urinary bladder. Abnormal lesions and all tissues collected from animals at sacrifice, animals in the high-concentration and VC groups, and animals found dead or accidentally killed (tissue integrity permitting) were further processed to slides, stained with hematoxylin and eosin, and examined microscopically.

### Statistical analysis

Multiple comparison tests were conducted for the different dose groups. The homogeneity of variance was examined using the Bartlett’s test. Homogeneous data were analyzed using analysis of variance, and the significance of inter-group differences was analyzed using Dunnett’s test. Heterogeneous data were analyzed using the Kruskal–Wallis test, and the significance of inter-group differences between the control and treatment groups was assessed using Dunn’s Rank Sum Test.

To compare the control and recovery groups, the data were analyzed for homogeneity of variance using the F test. Homogeneous data were analyzed using the t test, and the significant difference between the control and recovery groups was assessed using Dunnett’s test. Heterogeneous data were analyzed using the Kruskal–Wallis test, and significant differences between the control and recovery groups were assessed using Dunn’s Rank Sum Test (Table 3).

**Table 3:** Microscopic observation results of male and female mice after administration of human NK cells

Dose (cells/head)	Male SCID mice					Female SCID mice				
	VC	5 × 10.^6^	2 × 10.^7^	VC (R)	2 × 10.^7^ (R)	VC	5 × 10.^6^	2 × 10.^7^	VC (R)	2 × 10.^7^ (R)
Number examined	10	10	9	6	6	10	9	10	6	6
Tissue with diagnoses										
Heart										
Infiltration, mononuclear cell	0	0	1	2	0	2	1	1	0	0
Mineralization	1	3	2	2	1	4	2	2	0	1
Injection site(s)										
Fibroplasia	1	0	1	1	0	0	2	1	0	0
Hemorrhage	0	1	0	0	0	0	1	1	0	0
Infiltration, mixed cell	2	1	4	0	0	2	0	5	0	0
Infiltration, mononuclear cell	6	5	4	2	1	6	6	4	1	1
Necrosis, fibrinoid	2	4	1	0	0	0	0	2	0	0
Ulcerative dermatitis	1	0	0	0	0	0	0	1	0	0
Hemolymph node										
Lymphoma	0	0	0	0	0	1	0	1	0	0

*R* recovery period, *SCID* severely combined immunodeficient, *VC* vehicle control

## Results

### Characterization of GMP-expanded NK cells

We expanded NK cells from healthy donors by culturing PBMCs in the presence of IL-2 for 17 days in a GMP-compliant facility. On day 17, the products of GMP-expanded human NK cells were composed of highly enriched CD56^+^ (98.94 ± 0.01%) NK cells with minimal contamination by CD3^+^ T cells (0.84 ± 0.01%), CD14^+^ monocytes (0.13 ± 0.11%) or CD20^+^ B cells (0.51 ± 0.01%). The cytotoxic activity of the expanded NK cell population was evaluated against K562, the human immortalized myelogenous leukemia cell line (E:T ratio = 10:1). In cytotoxicity assays against the K562 cell line, expanded NK cells showed increased cytolytic activity compared with freshly isolated NK cells (data not shown).

**Fig. 1:**
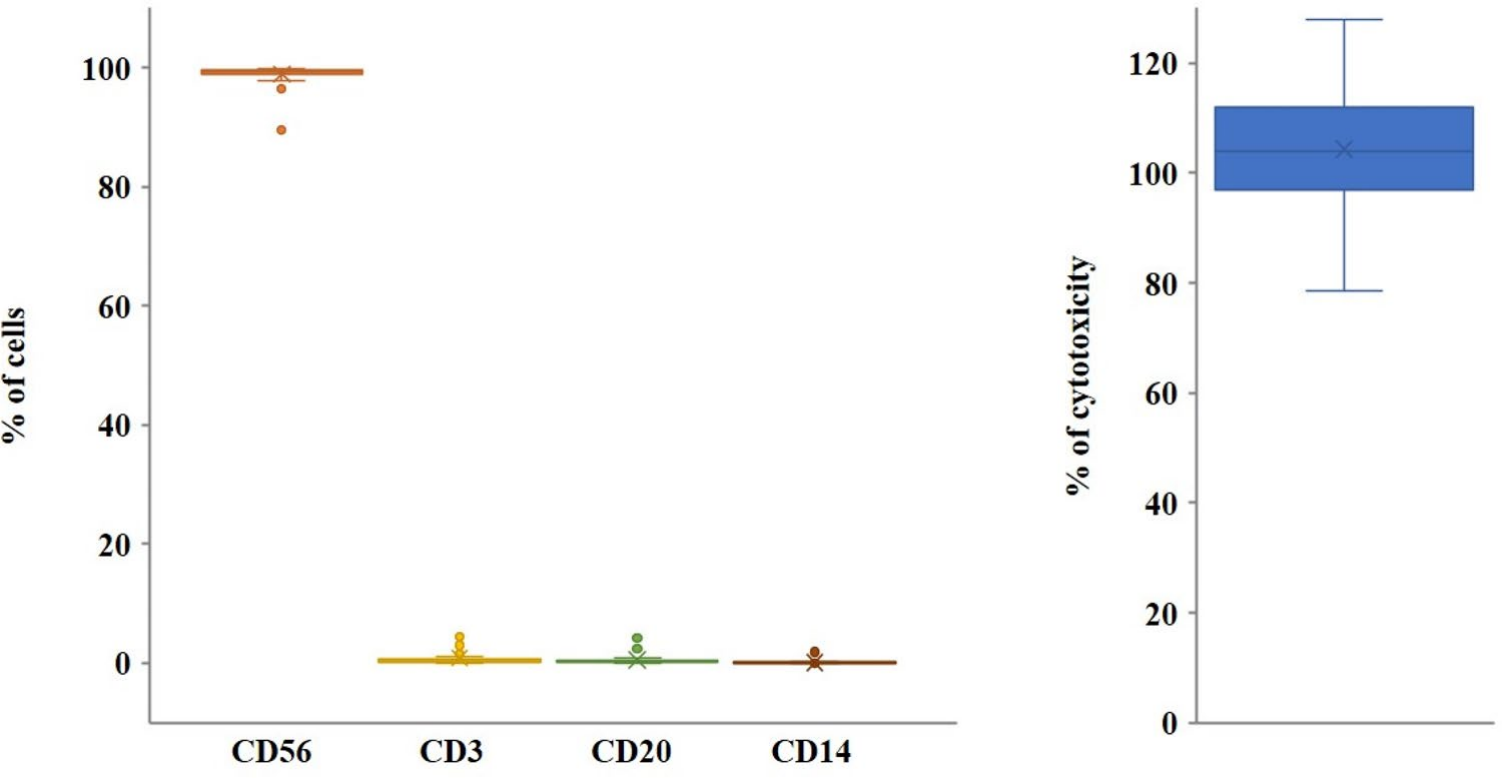
Characterization of GMP-expanded NK cells. The percent of CD56^+^, CD3^+^, CD20^+^, CD14 ^+^ cells were analyzed using flow cytometry. Cytotoxicity of NK cells against K562 cells was evaluated. The effector:target ratio was 10:1

Criteria for the release of human NK cells (SuperNK^®^, NKMAX Co., Ltd.) for animal use included the absence of microbial contamination (bacteria, fungus, virus, and mycoplasma), viability greater than 80% when assessed using a trypan blue exclusion assay, cytotoxicity greater than 50% against K562 target cells at the effector to target cell ratio of 10:1, endotoxin level equal to or less than 0.5 EU/mL, and immune phenotyping via flow cytometric analysis, proving expression of NK cell marker (CD56^+^/CD3^-^) (80% or more) and absence of CD14, CD34, and CD45 (5% or less) (Fig. 1).

### Single dose toxicity

**Fig. 2:**
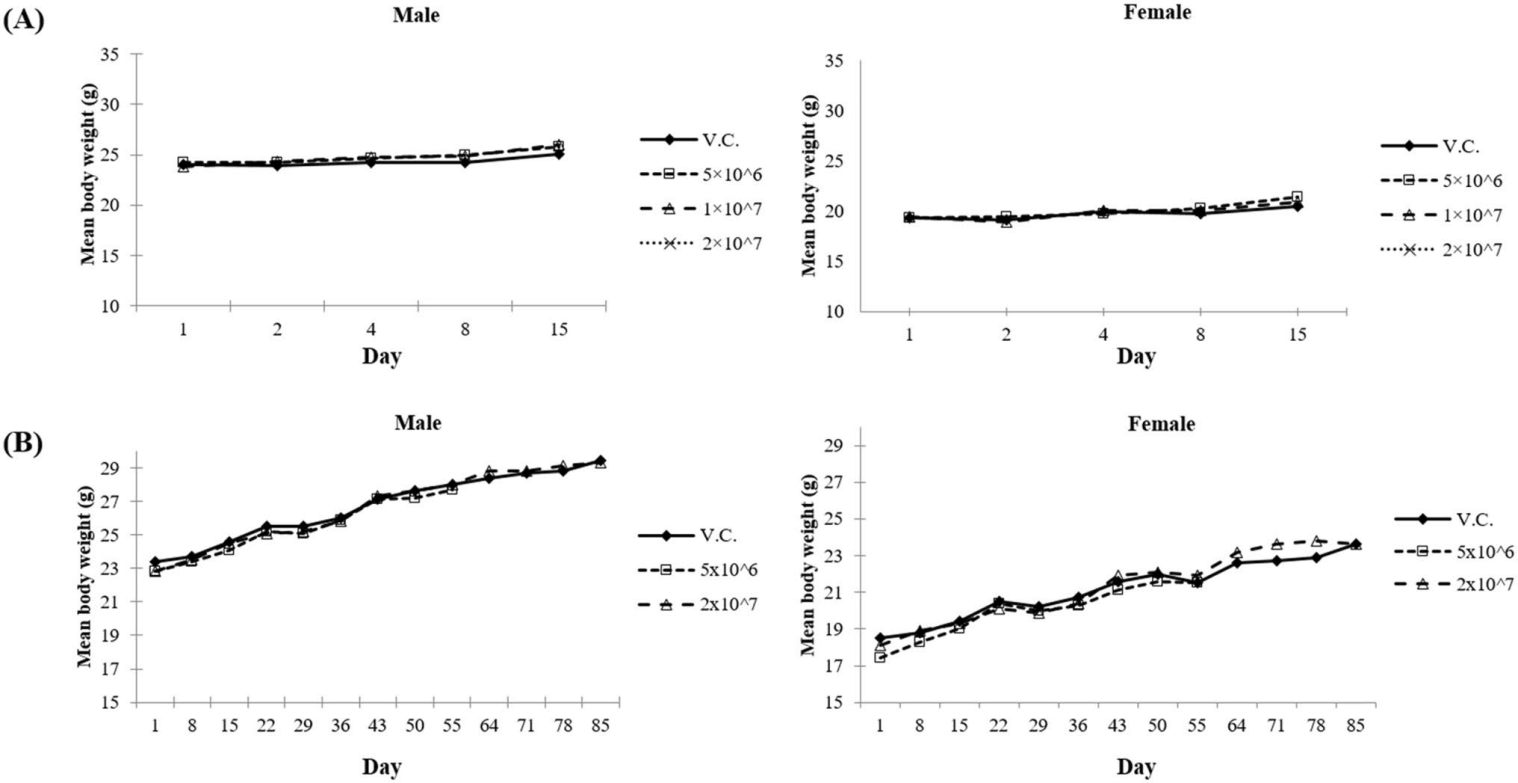
Body weight changes after single (**a**) and 24 times repeated (**b**) administration of NKMAX Cell Therapy Product

No treatment-related mortality or changes in clinical signs, body weight, or macroscopic findings were observed (Fig. 2A). Therefore, the approximate lethal dose of human NK cells was considered to be more than 2 × 10^7^ cells/head.

### Eight-week repeated dose toxicity study

There were no treatment-related toxicities, with respect to changes in clinical signs, body weight, food consumption, ophthalmology, clinical chemistry, urinalysis, macroscopic findings, and microscopic findings in the preliminary two weeks repeated dose toxicity study (unpublished data).

One female mouse in the VC group died during the recovery period on day 24 due to incidental lymphoma. One male in the high-dose group at day 8 and one female in the high-dose group at day 6 died, and no significant change was observed during microscopic examination. Therefore, these deaths were not considered to be related to human NK cells, although the cause of death was not determined.

Loss of tail and/or skin coloration in the lower tail region were found in some animals. These findings were considered to be accidental changes due to the administration method, as they were observed in a dose-dependent manner in certain animals, including those in the VC group.

Statistically significant increase in weight was observed in female animals in the low-dose and high-dose groups (maximum 1.59 times and 1.27 times, respectively), when compared with that in the VC group. However, there were no body weight changes or human NK cell-related toxicological observations and findings (Fig. 2B). Therefore, it was not considered to be toxicologically significant. A statistically significant decrease in food consumption was observed in male animals in the low-dose and high-dose groups during the treatment period (maximum 83% and 87%, respectively, compared with that in the VC group). However, the weight was recovered during the recovery period. Statistically significant increase in food consumption was sporadically observed in female animals in the low-dose and high-dose groups during the treatment and recovery periods (maximum 1.15- and 1.18 times, respectively, compared with that in the VC group). There was no dose-dependent consistency within sex and body weight changes; therefore, it was considered to be not toxicologically significant.

Absolute monocyte was decreased in females in the high-dose group (55% of control), but it recovered after a 4-week recovery period. This change was not considered to be related to human NK cells but was incidental, as this change did not exhibit any microscopic correlation and was not observed in males. Other statistically significant changes in the recovery groups were not considered human NK cell-related, as these were not observed in the main groups. A decrease in total cholesterol level was observed in males in the low-dose and high-dose groups (89% of control) and a decrease in albumin levels was observed in males in the high-dose group (93% of the control group). In addition, the level of gamma glutamyl transpeptidase was increased in females in the high-dose group (2.71 times over control). These changes were not considered human NK cell-related but were considered incidental because these changes did not show consistency between males and females, and there were no microscopic correlations. Other statistically significant changes in the recovery groups were not considered human NK cell-related, as these were not observed in the main groups. An increase in relative kidney weight was observed in the main group of females in the high-dose group (approximately 1.07 times the body weight and 1.09 times the brain weight, at necropsy) when compared to that in the VC group. However, this change was not considered human NK cell-related, but it was considered incidental, as there were no microscopic correlations. Other changes in relative organ weight in the recovery group were not considered to be human NK cell-related but were considered incidental.

**Fig. 3:**
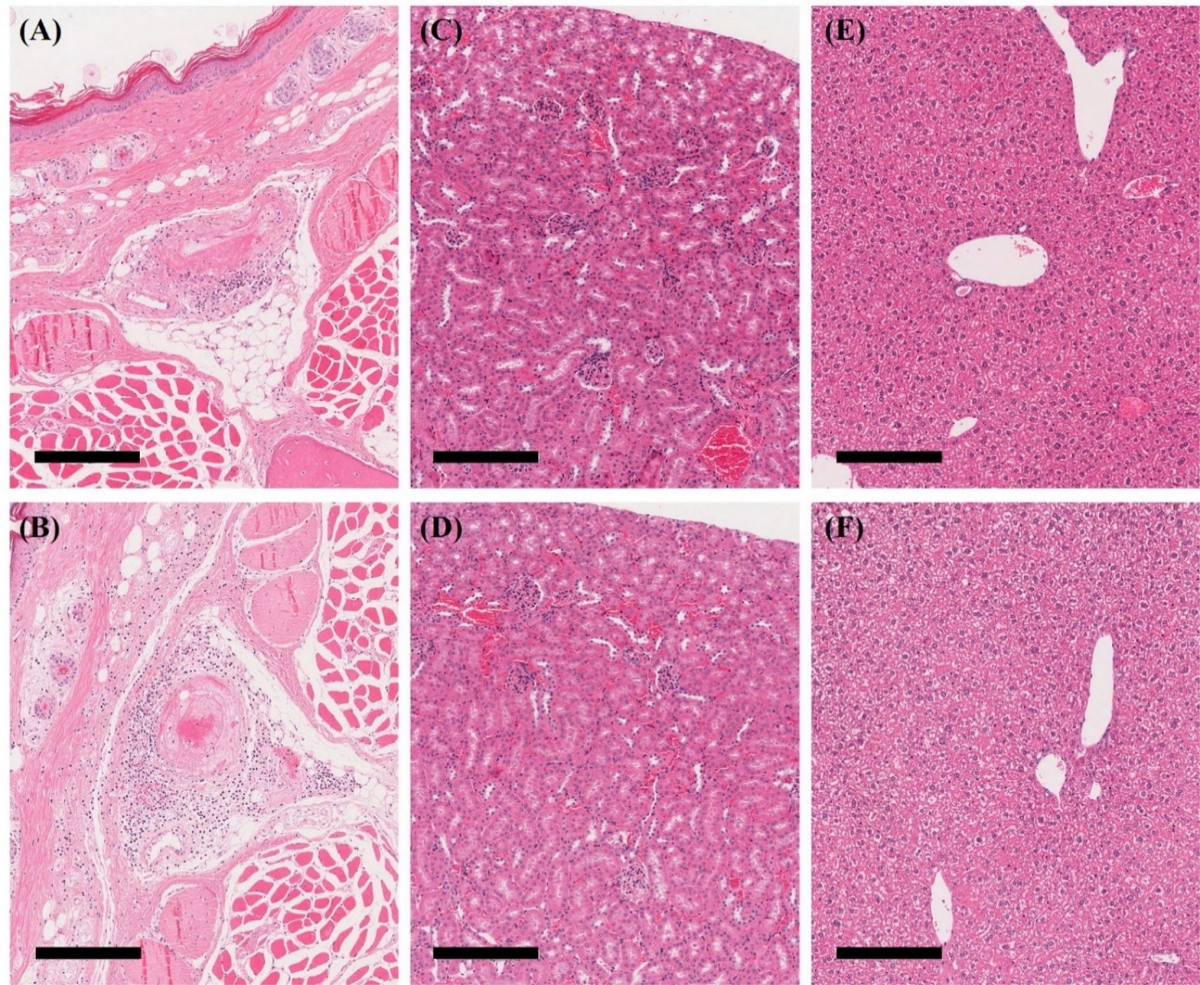
Histopathological changes of injection site (**a**, **b**), kidney (**c**, **d**) and liver (**e**, **f**) after 24 times repeated administration of NKMAX Cell Therapy Product. Panel **a**, **c**, **e** VC group; panel **b**, **d**, **f** 5 × 10^6^ cells/head group. Scale bar 200 μm

Necropsy revealed ulceration at the injection site (tail), observed in one male in the VC group and one female in the low-and high-dose groups. These findings were consistent with microscopic ulcerative dermatitis, which was considered to be induced by the process of injection into the tail vein. Microscopic observation revealed mononuclear cell infiltration in the perivascular area, mixed cell infiltration accompanied by neutrophil infiltration and mononuclear cell infiltration, and fibrinoid necrosis in both VC and treatment groups, at the injection site (Fig. 3). In addition, ulcerative dermatitis was observed in both VC and treatment groups. These changes were not considered human NK cell-related but local damage caused by the dosing procedure. In the heart, mineralization in the epicardium was observed in both the VC and treatment groups. This can be considered a spontaneous change that often occurs in SCID mice [15]. Lymphomas were also observed in some animals in the treatment group. However, this change was not considered human NK cell-related, as one dead animal in the VC group also showed lymphoma, which often occurs in SCID mice [16]. Other microscopic findings were considered incidental or spontaneous, given their low incidence and severity. There were no test item-related changes in ophthalmology or urinalysis.

Therefore, human NK cell-related findings were not observed in any of the test parameters. The no observed adverse effect level (NOAEL) of human NK cells was considered to be 2 × 10^7^ cells/head for both male and female SCID mice.

### Method validation for qPCR

Samples for the calibration curve were prepared with DNA extracted from the NKMAX Cell Therapy Product as the reference standard DNA. The calibration curve consisted of seven concentrations (0.005, 0.01, 0.05, 0.1, 1, 10, and 50 ng), and linearity was assessed using three repeated runs. The correlation coefficient (R^2^) of the calibration curve was 0.9973.

Specificity was conducted to confirm whether the reference standard DNA was specifically reacted compared to the DNA extracted from tissues (brain and spleen) other than the blank matrix. The amount of DNA used in each well was approximately 100 ng. As a result of the analyses in reference standard, no template control (NTC, mixture control without spiked reference DNA), and blank (BLK), the Ct value of DNA extracted from brain and spleen was higher than lower limit of quantitation (LLOQ) in NTC and BLK. Accuracy and precision were evaluated by analyzing quality control (QC) samples spiked with genomic DNA from SCID mice. QC samples were prepared with LLOQ (0.005 ng), low QC (0.015 ng), middle QC (0.5 ng), and high QC (40 ng). Accuracy and precision were determined as % relative error (%RE) and % relative standard deviation (%RSD) of each QC concentration level, respectively. Results from the analysis showed that both %RE and %RSD were within 20% (except 25% at LLOQ).

Quantification per tissue was conducted to determine if genomic DNA extracted from certain tissues had an effect on the linearity range by quantifying the calibration curve with reference standard DNA spiked with genomic DNA extracted from the brain and spleen of SCID mice. The coefficients of correlation (R^2^) of the calibration curves for the brain and spleen were 0.997 and 0.998, respectively.

Overall, these findings confirmed that this method was accurate, precise, and reproducible to quantify the human *Alu* gene in the tissue of SCID mice.

### Biodistribution

**Fig. 4:**
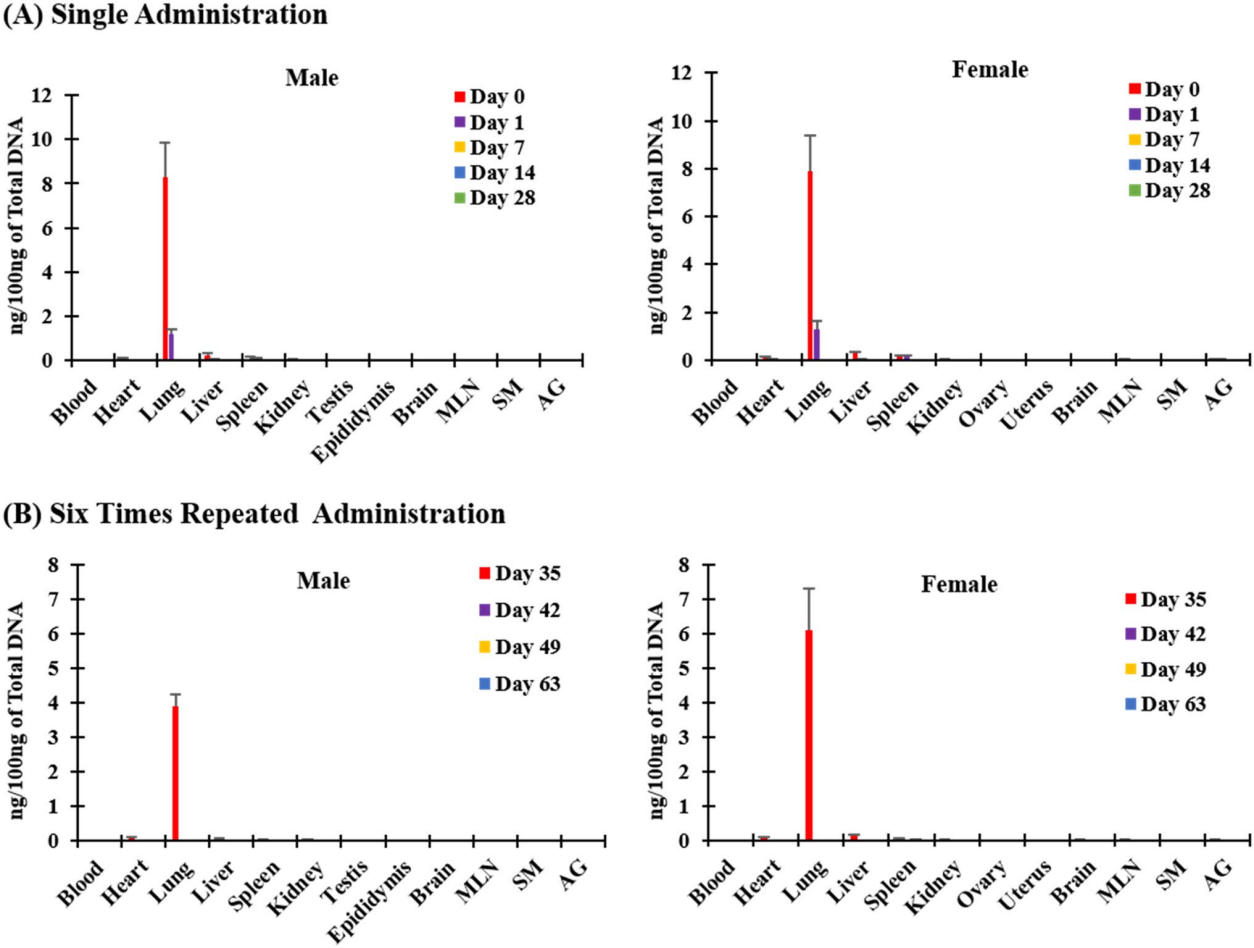
Biodistribution result after single (**a**) and repeated (six times) (**b**) administration of NKMAX Cell Therapy Product. NKMAX Cell Therapy Product was mainly distributed in the lungs, and a small number of the cells were detected in the liver, heart, spleen, and kidney

Results from the qPCR analysis showed that NKMAX Cell Therapy Product was mainly distributed in the lungs, and a small amount of NKMAX Cell Therapy Product was detected in the liver, heart, spleen, and kidney after single administration. After repeated administration, NKMAX Cell Therapy Product was observed in the lung, while a small amount was detected in liver, heart, spleen, kidney, adrenal glands (Fig. 4). NKMAX cell therapy product was completely eliminated in all organs of single-dosed animals on day 7, after dosing. Complete elimination was confirmed on day 64 (28 days after the last dose) and day 43 (7 days after the last dosing) in repeated dosed male and female animals, respectively.

Therefore, NKMAX cell therapy product was mainly distributed in the lung and completely eliminated 7 days after single administration (day 8) in both sex animals and on 28 day after the last dose (day 64) in males and 7 days after the last dosing (day 43) in females after six administrations.

## Discussion

Considering that the population of NK cells in PBMCs is in the range of 5–20%, GMP-based manufacturing, focused on expansion and purity of NK cells, is an important factor for the intended therapeutic application. Several strategies have been attempted to isolate and expand primary NK cells from PBMCs, but only a few strategies have been succeeded that follow the strict requirements of GMP [17–19]. Although it has been reported that NK cells are non-toxic and safe as therapeutic cell products, and non-hematological toxicity may be reduced by pretreatment with chemotherapy and radiation, cytokine-related toxicity may occur because of the concomitant administration of cytokines and related conjugates [20]. Although cytokines are essential for the expansion of NK cells, severe adverse effects, such as organ dysfunction, have been reported [21]. Furthermore, there are safety concerns when using feeder cells because they may contaminate the final therapeutic cell product, and this is also why the purity of NK cells is important from the perspective of safety [22]. In addition, the possibility of damage to the immune-privileged site was raised by the low expression of MHC-I in NK cell-enhanced immunotherapy [23].

In the present study, we evaluated the general toxicity and biodistribution of human NK cells treated with IL-2 and KL-1 (human erythroblastoid cell line) as feeder cells. NK cells produced in the GMP facility showed a high and sufficient purity of 98.9% and functional capabilities (Fig. 1). In the toxicity study, there were no test item-related toxicological changes after either single or repeated administration, and the NOAEL of human NK cells was considered to be 2 × 10^7^ cells/head or higher for both male and female SCID mice.

In a method validation study using qPCR, 0.005 ng of LLOQ showed sensitivity to detect up to one human cell. Using this method, biodistribution kinetics showed that administered NK cells initially accumulated in the lungs due to their cell size and receptor-mediated adhesion, and at later time points, they disseminated to other organs (including heart, lung, liver, spleen, brain, mesenteric lymph node, and adrenal gland) and completely disappeared by day 13 [24–26]. This finding suggested that the tumorigenicity potential of NK cells is low.

In conclusion, human NK cell transplantation to SCID mice showed no toxicity and were mainly distributed in the lung and completely disappeared from the body over time after single or repeated intravenous administration. The preclinical efficacy and toxicity of human NK cells in various tumor or humanized models need to be determined in future studies.

## References

[1] Hu Y, Tian Z, Zhang C (2019). Natural killer cell-based immunotherapy for cancer: advances and prospects. Engineering.

[2] Cheng M, Chen Y, Xiao W, Sun R, Tian Z (2013). NK cell-based immunotherapy for malignant diseases. Cell Mol Immunol.

[3] Kim S, Iizuka K, Aguila HL, Weissman IL, Yokoyama WM (2000) In vivo natural killer cell activities revealed by natural killer cell-deficient mice. Proc Natl Acad Sci USA 97:2731–2736. 10.1073/pnas.050588297 10.1073/pnas.050588297 PMC15998 10694580

[4] Smyth MJ, Hayakawa Y, Takeda K, Yagita H (2002). New aspects of natural-killer-cell surveillance and therapy of cancer. Nat Rev Cancer.

[5] Handgretinger R, Lang P, André MC (2016). Exploitation of natural killer cells for the treatment of acute leukemia. Blood.

[6] He Y, Tian Z (2017). NK cell education via nonclassical MHC and non-MHC ligands. Cell Mol Immunol.

[7] Denman CJ, Senyukov VV, Somanchi SS, Phatarpekar PV, Kopp LM, Johnson JL (2012). Membrane-bound IL-21 promotes sustained ex vivo proliferation of human natural killer cells. PLoS One.

[8] Luevano M, Daryouzeh M, Alnabhan R, Querol S, Khakoo S, Madrigal A (2012). The unique profile of cord blood natural killer cells balances incomplete maturation and effective killing function upon activation. Hum Immunol.

[9] Dalle JH, Menezes J, Wagner E, Blagdon M, Champagne J, Champagne MA (2005). Characterization of cord blood natural killer cells: implications for transplantation and neonatal infections. Pediatr Res.

[10] Rengasamy M, Gupta PK, Kolkundkar U, Singh G, Balasubramanian S, Sundarraj S (2016). Preclinical safety & toxicity evaluation of pooled, allogeneic human bone marrow-derived mesenchymal stromal cells. Indian J Med Res.

[11] Geller MA, Cooley S, Judson PL, Ghebre R, Carson LF, Argenta PA (2011). A phase II study of allogeneic natural killer cell therapy to treat patients with recurrent ovarian and breast cancer. Cytotherapy.

[12] Childs RW, Carlsten M (2015). Therapeutic approaches to enhance natural killer cell cytotoxicity against cancer: the force awakens. Nat Rev Drug Discov.

[13] Lim SA, Kim TJ, Lee JE, Sonn CH, Kim K, Kim J (2013). Ex vivo expansion of highly cytotoxic human NK cells by cocultivation with irradiated tumor cells for adoptive immunotherapy. Cancer Res.

[14] Nicklas JA, Buel E (2003). Development of an Alu-based, real-time PCR method for quantitation of human DNA in forensic samples. J Forensic Sci.

[15] Raghunathan S, Reynolds CL, Stewart MD, Schwartz RJ, McConnell BK (2017). Spontaneous dystrophic cardiac calcinosis in CB-17 SCID mice. FASEB J.

[16] Kato C, Fujii E, Chen YJ, Endaya BB, Matsubara K, Suzuki M (2009). Spontaneous thymic lymphomas in the non-obese diabetic/Shi-scid, IL-2R gamma null mouse. Lab Anim.

[17] Brehm C, Huenecke S, Quaiser A, Esser R, Bremm M, Kloess S (2011). IL-2 stimulated but not unstimulated NK cells induce selective disappearance of peripheral blood cells: concomitant results to a phase I/II study. PLoS One.

[18] Koehl U, Brehm C, Huenecke S, Zimmermann SY, Kloess S, Bremm M, Ullrich E, Soerensen J, Quaiser A, Erben S, Wunram C, Gardlowski T, Auth E, Tonn T, Seidl C, Meyer-Monard S, Stern M, Passweg J, Klingebiel T, Bader P, Schwabe D, Esser R (2013). Clinical grade purification and expansion of NK cell products for an optimized manufacturing protocol. Front Oncol.

[19] Linn YC, Yong HX, Niam M, Lim TJ, Chu S, Choong A (2012). A phase I/II clinical trial of autologous cytokine-induced killer cells as adjuvant immunotherapy for acute and chronic myeloid leukemia in clinical remission. Cytotherapy.

[20] Suen WC-W, Lee WY-W, Leung KT, Pan XH, Li G (2018). Natural killer cell-based cancer immunotherapy: a review on 10 years completed clinical trials. Cancer Investig.

[21] Rosenberg SA, Lotze MT, Yang JC, Aebersold PM, Linehan WM, Seipp CA (1989). Experience with the use of high-dose interleukin-2 in the treatment of 652 cancer patients. Ann Surg.

[22] Oh S, Lee JH, Kwack K, Choi SW (2019). Natural killer cell therapy: a new treatment paradigm for solid tumors. Cancers.

[23] Bolourian A, Mojtahedi Z (2017). Possible damage to immune-privileged sites in natural killer cell therapy in cancer patients: side effects of natural killer cell therapy. Immunotherapy.

[24] Gao J, Dennis JE, Muzic RF, Lundberg M, Caplan AI (2001). The dynamic in vivo distribution of bone marrow-derived mesenchymal stem cells after infusion. Cells Tissues Organs.

[25] Meyerrose TE, De Ugarte DA, Hofling AA, Herrbrich PE, Cordonnier TD, Shultz LD (2007). In vivo distribution of human adipose-derived mesenchymal stem cells in novel xenotransplantation models. Stem Cells.

[26] Fischer UM, Harting MT, Jimenez F, Monzon-Posadas WO, Xue H, Savitz SI (2009). Pulmonary passage is a major obstacle for intravenous stem cell delivery: the pulmonary first-pass effect. Stem Cells Dev.

